# Exposure therapy for PTSD during pregnancy: a feasibility, acceptability, and case series study of Narrative Exposure Therapy (NET)

**DOI:** 10.1186/s40359-020-00503-4

**Published:** 2020-12-09

**Authors:** Natalie R. Stevens, Michelle L. Miller, Christina Soibatian, Caitlin Otwell, Anne K. Rufa, Danie J. Meyer, Madeleine U. Shalowitz

**Affiliations:** 1 Rush University Medical Center, 1645 West Jackson Blvd, Chicago, IL 60612 USA; 2 VA Greater Los Angeles Healthcare System, 11301 Wilshire Blvd, Los Angeles, CA 90073 USA; 3 Vivo International, P.O. Box, 5108D-78430 Konstanz, Germany

**Keywords:** Posttraumatic stress disorder (PTSD), Pregnancy, Exposure therapy, Narrative exposure therapy, Perinatal mental health

## Abstract

**Background:**

Prenatal posttraumatic stress disorder (PTSD) is a significant complication of pregnancy linked to increased risk of adverse perinatal outcomes. Although 1 in 5 pregnant trauma-exposed individuals have PTSD, most PTSD treatment trials exclude participants who are pregnant, and none focus on treatment specifically during pregnancy. Moreover, access to mental health treatment is particularly challenging in low-resource settings with high rates of trauma. This study examined implementation of Narrative Exposure Therapy (NET), a short-term evidence-based PTSD treatment, in an urban prenatal care setting. Partial telehealth delivery was used to increase accessibility. Study aims were to examine (a) feasibility, (b) acceptability, and (c) case-based treatment outcomes associated with NET participation.

**Method:**

Eight pregnant participants (median age = 27, median gestational week in pregnancy = 22.5) received up to six sessions of NET with partial telehealth delivery. PTSD and depression symptoms were assessed at pre-treatment intake (T1), at each session (T2), and 1-week post-treatment (T3). A multiple case study approach was used to examine recruitment and engagement, retention, treatment completion, treatment barriers, use of telehealth, participants’ experiences of treatment, and PTSD and depression symptoms.

**Results:**

Nine of the 16 participants (56%) who were invited to participate engaged in treatment, and one dropped out after the first session. Eight participants completed the minimum “dose” of 4 NET sessions (*N* = 8/9, 89%). Seven participants gave the highest ratings of treatment acceptability. The most frequently reported barriers to treatment were competing priorities of work and caring for other children. Pre-post treatment symptom measures revealed clinically meaningful change in PTSD severity for nearly all participants (7/8, 88%).

**Conclusions:**

Results suggest that a brief exposure therapy PTSD treatment can be successfully implemented during pregnancy, suggesting promising results for conducting a larger-scale investigation.

*Trial registration* ClinicalTrials.gov, NCT04525469. Registered 20 August 2020–Retrospectively registered, https://register.clinicaltrials.gov/prs/app/template/EditRecord.vm?epmode=View&listmode=Edit&uid=U00058T2&ts=3&sid=S000A59A&cx=-w1vnvn

Approximately 1 in 5 trauma-exposed individuals worldwide suffer from clinically significant symptoms of posttraumatic stress disorder (PTSD) during pregnancy [1]. Although we lack data demonstrating causality, pregnant individuals with PTSD are at increased risk of adverse perinatal outcomes, including preterm birth, low birthweight, obstetrical complications, and postpartum mood and anxiety disorders [2–5]. Interventions designed to reduce PTSD symptoms during pregnancy are extremely limited [6]. We are aware of only three psychological intervention studies that included prenatal PTSD symptoms as a clinical outcome. [7–10]. These intervention studies were acceptable to participants, feasible, and associated with improved perinatal outcomes [7–12]. However, these interventions are not intended specifically for the treatment of PTSD diagnosis, instead focusing on psychoeducation. Unfortunately, most treatment trials for PTSD exclude participants who are pregnant, and none specifically focus on this developmental period of heightened risk. The purpose of this feasibility and case series analysis is to report results of a short-term behavioral treatment for PTSD using Narrative Exposure Therapy (NET) for individuals who are currently pregnant [13].

The evidence for behavioral treatments for PTSD, including the gold standard exposure therapy (e.g. Prolonged Exposure (PE)) [14], is based on studies in which pregnancy is a criterion for exclusion [15]. As a result, PTSD in pregnancy is largely unassessed and remains untreated. This leads to a greater symptom burden for trauma-exposed pregnant individuals at a critical developmental juncture as well as additional, associated medical and psychological risk of pregnancy complications and poor postpartum outcomes associated with PTSD [16, 17]. If causal, disparities in exposure to traumatic stress may be linked to the stark racial disparities in maternal and infant morbidity and mortality [18, 19]. People of color living in low-resource communities are at increased risk of trauma both in childhood and adulthood, [20–22], are disproportionately affected by PTSD in pregnancy, and are less likely to receive mental health treatment compared to white populations [18, 23]. The absence of evidence-based clinical recommendations for the management and treatment of traumatic stress almost certainly has a differential, negative impact and likely contributes to racial/ethnic disparities in perinatal health. Our aim is to begin to build an evidence base for PTSD treatment for pregnant populations in general, while focusing on communities of color in particular. We examined the feasibility, acceptability, and treatment outcomes of a brief exposure therapy among eight pregnant participants who met criteria for PTSD.

Narrative Exposure Therapy (NET) [13] is an evidence-based PTSD treatment that uses a life-span approach to chronologize and contextualize formative life events in the treatment of cumulative or complex trauma, such as childhood abuse and intimate partner violence. NET is a manualized, short-term trauma therapy (4 to 12 sessions) that integrates exposure and testimony therapy to reconstruct an individual’s autobiographical memories of traumatic events [24]. NET is effective in reducing PTSD symptoms in as few as four sessions [25], a time commitment that is less than for other evidence-based PTSD treatments. Therefore, NET may be a particularly promising treatment to reduce PTSD symptoms in pregnant individuals with multiple lifetime exposures to traumatic events and currently undergoing a significant life change. Additionally, NET has no literacy requirement and has been found to be feasible and effective cross-culturally and in low-resource settings, including war zones, refugee camps, and other trauma-exposed communities [26, 27].

NET’s basis in testimony therapy is culturally relevant and acceptable among diverse non-white cultural groups [28–30]. Testimony therapy offers the opportunity to view the individual’s life story in the context of the greater community, an element particularly valued in African-American culture [31]. Ritualized in Black church traditions, testimony “allows Black people to find their healing by reconnecting with the deepest pain, as well as the deepest joy, that defines the personal and collective Black experience” [31, pg.9]. NET’s utilization of testimony has demonstrated efficacy across diverse cultures and geographies in more than 12 randomized controlled trials internationally, regardless of traumatic event type and severity [24, 32].

Taken together, these observations offer a strong case for investigating NET as a treatment for PTSD during pregnancy [33, 34] in a prenatal care setting in which collaboration with perinatal healthcare providers is facilitated through a shared electronic medical record and co-located behavioral health services. Situated within an academic medical center, our clinical setting primarily serves low-income, pregnant patients from the surrounding urban communities, most of whom (> 70%) identify as Black or Hispanic/Latina. To increase treatment accessibility and retention while navigating the known barriers to mental health treatment affecting this population, the current study delivered NET partially via telehealth. Telehealth provides a safe and effective medium for conducting exposure therapy [39] and increases the likelihood of completing treatment amidst treatment barriers (e.g., cost, transportation, time, etc.) without sacrificing treatment efficacy [35–39].

The current, open pilot study was designed to investigate the following aims: (1) feasibility, (2) acceptability, and (3) treatment outcomes associated with receiving brief NET (up to six sessions) with partial telehealth delivery (i.e., telehealth offered for the last two sessions). To determine feasibility, we evaluated: (a) the proportion of pregnant patients who met eligibility criteria that engaged in treatment, (b) the proportion of participants who received the minimum dose of NET, and (c) barriers to treatment retention. To determine acceptability, we utilized (a) a quantitative analysis of NET acceptability measures and (b) qualitative feedback from participants. To determine if NET participation is associated with clinically-meaningful reductions in symptoms, we assess pre-post treatment symptom changes for both depressive and PTSD symptoms, given the frequent comorbidity of PTSD and depression [40].

## Methods

### Design

We conducted an open pilot feasibility study offering NET to pregnant patients who were in the second trimester of pregnancy in an urban academic medical center in the Midwestern United States. Using a multiple case study approach, each individual participant’s data was examined utilizing different data sources to capture information on recruitment and engagement, retention, treatment completion, barriers to completing the sessions, use of telehealth, qualitative evaluations of NET, and self-reported PTSD and depression symptoms. In this paper, we describe data collected at a pre-treatment intake session (T1) and at a 1-week post-treatment assessment (T3), as well as symptom monitoring at each session (T2).

### Participants

**Fig. 1:**
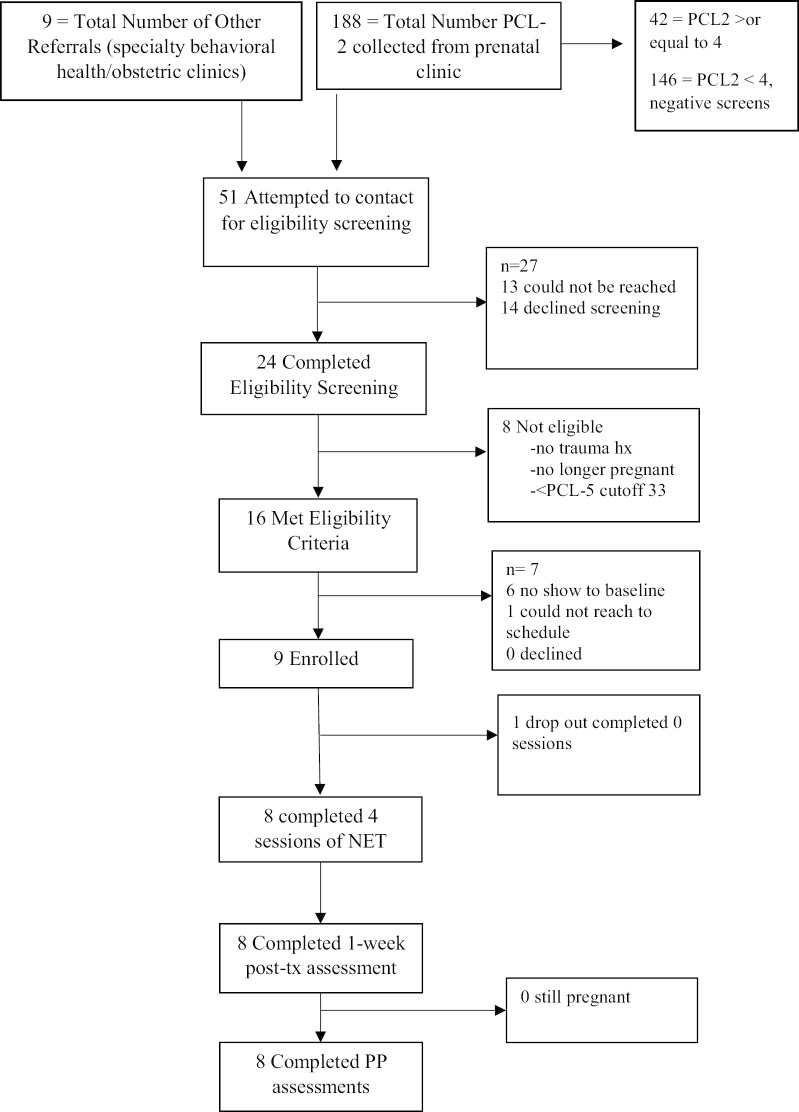
Participant Flow (CONSORT) Diagram

Participants in this case series analysis were eight pregnant patients who received at least 4 sessions of NET (See Fig. 1). The median age was 27 years (range 19–35). Participants described their race as Black (n = 3), Asian (n = 1), or white (n = 1) and three participants described their ethnicity as Hispanic. Five participants described their gender identity as female (data were missing for 3 participants). These three participants volunteered using she/her pronouns during the course of treatment, however. Six participants reported previous pregnancies and births. Four participants reported traumas related to prior pregnancies, including one who experienced a traumatic childbirth, two who experienced miscarriage (i.e., loss of pregnancy before 20 gestational weeks), and one who experienced both a neonatal death of twins born at 27 gestational weeks and later a stillbirth/fetal death (i.e., after 20 gestational weeks). The median number of children was 2 (range 1–2). Five of the participants were either married or living with a partner. Three participants had received counseling or psychotherapy in the past and one participant had a counselor whom she saw monthly for supportive counseling. All participants had a probable diagnosis of PTSD, as indicated by a lifetime history of at least one traumatic event on the Life Events Checklist and a score of 33 or higher on the PTSD Checklist for DSM-5 (PCL-5) [41].

### Procedures

#### Eligibility criteria

We used a purposive sampling approach to reach our target group: Adults in their first or second trimesters of pregnancy (before 28 gestational weeks) who met criteria for a probable diagnosis of PTSD based on telephone interview. Pregnant patients who were at more than 28 weeks gestation were excluded to minimize the likelihood that NET sessions would not be completed if birth occurred early. Inclusion criteria were (a) age 18 years or older, (b) proficient in English; (c) history of a DSM-5 Criterion-A trauma at least 3 months prior to the current pregnancy; (d) PCL-5 score ≥ 33. Potential participants were excluded on the basis of the following: (a) > 28 weeks pregnant at time of eligibility interview; (b) current involvement of the legal system related to the trauma; (c) lifetime history of psychotic or manic symptoms; (d) significant cognitive impairment or intellectual disability; (e) current participation in trauma-focused psychotherapy; (f) an unstable dose of psychiatric medications (medications must be stable for a period of at least 6 weeks); (g) current significant suicidal ideation; or (h) current severe dissociative symptoms.

#### Recruitment

We recruited participants between the months of May–October 2019 from prenatal clinics in an academic medical center in which approximately 75% of patients receive Medicaid and/or public assistance. The majority of this patient population resides in the surrounding urban community with a demographic of ≥ 50% Black/African-American and 35% Hispanic/Latinx. The largest of the obstetric clinics serves more than 1200 pregnant patients annually. We received potential participant referrals to the study one of three ways. First, one of the obstetrics clinics routinely screened for PTSD by asking patients to complete the 2-item PTSD Symptom Checklist (PCL-2) [42] at their first prenatal appointment. Providers offered those who endorsed probable symptoms of PTSD (PCL-2 ≥ 4) information about the study and an invitation to complete an eligibility interview. Second, prenatal care providers in other obstetric clinics at the medical center or within its affiliated community-based sites serving pregnant and parenting individuals introduced the study. Third, pregnant patients were recruited to the study if they were referred to the medical center’s specialty psychotherapy clinic for treatment of perinatal mood, anxiety, or trauma-related disorders. The clinic’s intake coordinator informed potentially qualifying patients about the study, and the study coordinator contacted interested patients to complete the eligibility interview.

Trained research assistants (study staff) conducted the 30-min eligibility interviews by telephone to determine whether interested patients met criteria for inclusion in the study. Research assistants were not involved with conducting the NET treatment. During the eligibility telephone interview, a probable PTSD diagnosis was determined using the Life Events Checklist (LEC) to assess history of at least one Criterion A traumatic event and PCL-5 score ≥ 33, along with meeting other inclusion and exclusion criteria. NET was offered to all participants based on the results of their eligibility interview, regardless of whether their PCL-5 symptom scores changed or improved by the initial intake session. All study procedures were approved by the University’s Institutional Review Board.

#### Treatment setting

All NET sessions, excluding the telehealth sessions, were conducted within an outpatient psychotherapy clinic located within the medical center, co-located with the prenatal clinics where participants were recruited. The clinic is staffed and supervised by licensed clinical psychologists and in direct consultation with psychiatrists and psychiatric nurse practitioners for psychiatric medication needs and assessment of acute crises. Prior to completion of NET sessions, therapists assessed participants’ needs and desire for ongoing counseling, psychotherapy, or psychiatric care services. Participants desiring referral to a clinic therapist following completion of NET received referrals and were encouraged to pursue treatment while remaining in the study. Therapists maintained care of their individual participants by reviewing their follow up assessments and addressing psychosocial issues on an as-needed basis through one-month postpartum.

#### Conducting the treatment

Following determination of study eligibility, each participant met with a trained therapist to complete the informed consent process, and individual treatment began immediately thereafter. Participants remained paired with the same therapist throughout treatment to ensure continuity of care. The intake session lasted approximately 2 h and each subsequent session lasted between 90 and 120 min. Sessions were scheduled during extended business hours between 8am and 7 pm Monday through Friday. Data were collected at the initial intake session (T1), before each of the NET treatment sessions (T2), and one week following completion of the final NET session (T3). Additional follow up assessments were conducted one month after the final NET session and again one month following the birth; however this paper will report only on data from pre-treatment (T1) to immediately post-treatment (T3).

At intake (T1), therapists conducted the informed consent process and a history of lifetime traumatic events at T1, which was used as the basis for the lifeline exercise and NET exposures. Participants also provided demographic information including age, race, ethnicity, employment status, education, and marital status. Sociodemographic barriers to engagement, current medications, medical and obstetrical history, full mental health treatment history, depressive symptoms, PTSD symptoms, and pre-treatment expectations were also assessed. Before each NET treatment session (T2), participants completed measures of PTSD and depression symptoms. One week following treatment completion (T3), participants completed measures of PTSD and depression symptoms, treatment evaluation, and acceptability of NET via electronic data self-report. Table 1 summarizes session objectives and activities as well as measures administered at each time point.

**Table 1:** Summary of study activities, objectives, and structure

Time point	Measures	Session objectives and activities	Platform
Eligibility	Screening Form PCL-5 C-SSRS CAPS-5 (Dissociation)	Determine individual’s eligibility for study	Structured Interview
Session 1	Demographics Pre-Tx Expectations PCL-5 + LEC EPDS	Psychoeducation of trauma and PTSD Laying out the *Lifeline* of significant life events “**stones**” = traumas “**candles**” = losses/grief “**flowers**” = positive events “**sticks**” = harm caused to others	In-person
Session 2	PCL-5 EPDS	First exposure session	In-person
Sessions 3–4	PCL-5 EPDS	Subsequent exposure sessions	In-person
Session 5	PCL-5 EPDS	Final exposure session	Telehealth
Session 6	PCL-5 EPDS	Final reading of the full trauma narrative	Telehealth
1-Week Post-Tx	PCL-5 EPDS Participant Satisfaction NAQ	Assessment of psychopathology symptoms and treatment evaluation	Self-report Questionnaire

*Note*: 1-Week Post-Tx = one week after last treatment session; PCL-5 = PTSD Symptom Checklist for DSM-5; C-SSRS = Columbia–Suicide Severity Rating Scale; CAPS-5 = Clinician-Administered PTSD Scale for DSM–5, dissociative items only; Pre-Tx Expectations = pre-treatment expectations; LEC = Life Events Checklist for DSM-5; EPDS = Edinburgh Postnatal Depression Scale; NAQ = NET Acceptability Questionnaire

#### NET treatment

NET comprises four distinct parts: (1) assessment of lifetime trauma history and PTSD symptoms and diagnosis, (2) psychoeducation about trauma and PTSD, (3) laying the lifeline (using symbolic materials to chronologically represent traumatic events), (4) exposure sessions (constructing the trauma narrative of each event in the context of the life course). After laying the lifeline, the participant and therapist decide on the traumatic events (stones) and significant positive events (flowers) to focus on in subsequent exposure sessions. Once exposure sessions begin, the therapist and participant can also review multiple traumatic events in a single session. After each session the therapist transcribes a written narrative of the exposure and reads it aloud at the start of the next session to correct any errors or missing components. Participants receive copies of the narrative at the conclusion of treatment.

During exposure sessions, participants chronologically narrate the significant events of their life story. They are asked to describe traumatic events in detail and to slow the narration at the “hotspots,” or moments in which the fear response peaks. The therapist encourages the participant to reexperience the event while simultaneously maintaining the connection to the “here and now.” This combination supports the temporal anchoring of the trauma memory in the past and is critical to conducting exposure therapy effectively [13]. The goal is to reconstruct disorganized and fragmented autobiographical memory of traumatic events, integrating the cognitive, emotional and somatic information (e.g., heart rate, respiratory rate) about the traumas with the declarative knowledge about event place and time into a coherent narration.

#### Study treatment team & supervision

The NET treatment team consisted of four therapists: One licensed clinical psychologist who also served as the study director and primary clinical supervisor, two psychology postdoctoral fellows, and one licensed clinical professional counselor. All therapists completed the same NET training (12-h training course) in March 2019 conducted by a qualified NET trainer. Therapists were supervised throughout treatment via individual supervision with the NET trainer and group supervision with the entire study team. The NET trainer reviewed transcripts of the session narratives to ensure necessary components of the exposure were elicited from the session (i.e., sensory, cognition, emotion, body cues associated with the “hotspot”) and reviewed audio recorded sessions to ensure the exposure was conducted consistent with the manual format (i.e., slowing down narration at the “hotspot,” maintaining connection in the “here and now”). Group supervision was also used to practice role-play demonstrations of exposures, to review one another’s transcribed narratives, and to problem solve challenging clinical topics, such as helping participants recognize and navigate avoidance and symptoms of dissociation.

#### Session length

Following the initial intake session, each therapist-participant dyad collaborated to schedule the subsequent sessions per the proposed weekly schedule of four in-person clinic sessions followed by two telehealth sessions. The telehealth sessions occurred later in the protocol so that that intake and initial exposures sessions could be completed in person to assess safety risk factors and establish rapport. In prior NET research, the minimum treatment dose to achieve clinical benefit was considered four sessions [43, 44]. The number of sessions was thereby set a priori at six in order to balance the need for adequate treatment, increase the likelihood of treatment completion prior to the birth and reduce treatment burden for individuals already facing the challenge of the increased appointments and procedures associated with routine prenatal care.

#### Telehealth NET sessions

The final two NET sessions were delivered via telehealth to all participants. Partial telehealth delivery was included in the protocol to increase accessibility and retention in treatment and decrease barriers to care that can accompany in-person visits (e.g. transportation, childcare, etc.). We used a Health Insurance Portability and Accountability Act (HIPAA) compliant video-based telehealth platform, Vidyo [45], which can be accessed via mobile device using a free application (VidyoConnect) or on a tablet, laptop, or desktop computer. In cases of lack of access to a device or the internet, participants were informed that a smartphone with a prepaid 6-month data plan would be provided to them at no cost. The Vidyo platform allows for both the provider and patient to see, hear and speak one another through an encrypted video-based platform and for participants to receive treatment in a private location of their own choosing (usually in the home). Telehealth sessions are also conducted on weekdays during extended business hours while the therapist is in clinic to ensure access to clinic resources and direct supervision with the study director as needed.

#### Data collection

During treatment (T2), therapists record session progress notes in a structured form created for the study. The progress note structure ensures consistency in reporting and focused on giving a detailed account of the content, structure, and process of each NET session. Therapists record each session’s date and time, participants’ gestational age in weeks, and any medical complications of pregnancy. The treatment process includes participants’ level of engagement, barriers or stressors, focus of the session (i.e., lifeline, stone or flower exposures), participants’ response (i.e., change in distress), and a brief mental status examination including suicidal ideation. Decisions to alter or stop study procedures are also noted. Progress notes are kept in secure electronic files and are not entered into the participants’ electronic medical records. However, care coordination notes are recorded in the medical record to ensure continuity of care where appropriate. At T3, participants complete PTSD and depression symptom measures, as well as treatment satisfaction and acceptability measures. Additionally at T3, participants are asked what they liked and what they would change in a free-response format.

Study data are collected and managed using REDCap electronic data capture tools hosted at Rush [46, 47]. REDCap (Research Electronic Data Capture) is a secure, web-based software platform designed to support data capture for research studies, providing (1) an intuitive interface for validated data capture; (2) audit trails for tracking data manipulation and export procedures; (3) automated export procedures for seamless data downloads to common statistical packages; and (4) procedures for data integration and interoperability with external sources.

#### Measures

*Eligibility screening form* This study-specific form determines eligibility based on inclusion and criteria. Participants are asked about exposure to traumatic events, PTSD symptoms, current legal issues, pregnancy, complications, history of mania or psychosis, cognitive status, current and past mental health treatment, and psychiatric hospitalizations.

*Suicide risk* The Columbia–Suicide Severity Rating Scale (C-SSRS) is a clinician-administered interview that assesses suicidal ideation and suicidal behavior over the lifetime and within the last month [48]. This measure is a brief, thorough assessment of suicidal risk that has been found to be reliable and valid across populations [48, 49], including in pregnant and postpartum populations [50].

*Dissociation* The Clinician-Administered PTSD Scale for DSM–5 (CAPS-5) is the gold standard clinician-administered structured diagnostic interview for PTSD [51]. This measure has been extensively validated, including in minority samples [52] and determining the prevalence of PTSD symptoms in prenatal women [53]. Only the last two items from this measure were used to screen for dissociative symptoms.

*Demographics* This study-specific form assessed: age, relationship status, highest educational level, race, ethnicity, employment status, income, economic sufficiency, number of dependent children, common health problems, physical activity, and medications.

*Pre-treatment expectations* A study-specific 8-item measure was utilized to assess pre-treatment expectations. Qualitative items assessed interest in and concerns about treatment. Participants were asked to rate how much they agree with the following statements on a scale of 0 (completely disagree) to 10 (completely agree): beliefs in ability to heal from trauma, to heal from PTSD, for PTSD treatment to be successful, to improve in trauma symptoms, and to improve in trauma symptoms by the end of therapy.

*PTSD symptoms* The Posttraumatic Stress Disorder Checklist for DSM-5 (PCL-5) is a 20-item measure that assesses the presence of a Criterion A index trauma and all the clusters of symptoms consistent with a diagnosis of PTSD in the DSM-5 [41]. Symptoms are assessed with a 5-point Likert scale (0 = Not at all, 4 = Extremely). Higher scores indicate increased severity of PTSD symptoms. The Life Events Checklist (LEC-5) is a 17-item checklist is an additional component of the PCL-5 that assesses a range of potentially traumatic events that a participant may have experienced in their lifetime, which was used in conjunction with the PCL-5 at baseline. The PCL-5 and LEC component have shown good reliability and validity in pregnant samples [54, 55].

*Depressive symptoms* The Edinburgh Postnatal Depression Scale (EPDS) is one of the most well-validated assessments of the severity of perinatal depressive symptoms [56, 57]. The 10-item self-report measure assesses common symptoms of perinatal depression (sleep, appetite, feelings of guilt, depressed mood) using a 4-point Likert scale (0 = Not at all/Never, 4 = Yes, Most of the Time). Higher scores indicate increased severity of depressive symptoms.

*Participant satisfaction* Participants also completed a 5-item study-specific measure of how much benefit they derived from the treatment, the extent to which they still use their skills, the amount of behavior change post-treatment, and what they liked/disliked about the program. All items were summed and averaged.

*Acceptability* The NET Acceptability and Feasibility Questionnaire (NAQ) is a 10-item adapted measure of participant feedback regarding acceptability and feasibility of NET [58]. A 5-point scale (1 = ‘I don’t agree at all’ to 5 = ‘I totally agree’) is used to describe agreement with statements about NET (e.g. “I think NET is useful”, “I feel safe discussing traumatic events”). This questionnaire was piloted in focus groups for pregnant trauma-exposed adolescents and their partners’ attitudes about NET and was used with permission from the author [33].

#### Data analysis

*Feasibility* To be feasible, pregnant participants must be able to engage in treatment and complete a minimum number of treatment sessions. We examine the *engagement rate*, defined as those meeting eligibility criteria attending at least the initial intake session and completing the informed consent process, as well as the *retention rate*, defined as those who provided informed consent completing at least four NET sessions. Analysis consists of examining the proportions of both engagement and retention.

Additionally, we examine barriers to retention in treatment qualitatively by extracting information from therapists’ session records regarding factors that interfered with participants’ attendance at sessions, reasons for cancellation or rescheduling sessions, as well as factors that affected participants’ engagement in the session itself. Finally, participants’ gestational age in weeks is recorded and tracked at each session and assessment time point to examine the timing in pregnancy of when participants engaged in treatment and how long it took to complete treatment relative to the proposed 6-week treatment model.

*Acceptability* To be considered acceptable as a treatment, participants had to report pre-treatment that they had some belief that overcoming PTSD is both important and possible. Participants also answer qualitative questions assessing reasons for and concerns about participating in NET. Additionally, participants report post-treatment if the treatment they received is acceptable, if they understand how it works, and if they believe NET helped reduce their distress. To meet those benchmarks, we examine participant response measures of treatment expectations (T1) and NET acceptability (T3). For treatment expectations, we calculate the number of participants that endorse a 50% or higher chance of success in recovery and treatment on a 3-item study-specific questionnaire. To determine acceptability post-treatment, participants are asked how much they agreed with various statements about the usefulness, safety, and structure of NET. All items are summed and averaged, with a mean score of 3 or higher out of 5 considered acceptable. Additionally, participant responses to open-ended questions before starting and at the conclusion of treatment are collected and analyzed qualitatively reviewing raw responses case by case to determine whether participants viewed treatment as generally favorable.

*Preliminary treatment outcomes* To determine treatment outcomes, we calculated changes in PTSD and depression symptom scores from T1 to T3 for each participant. A 10-point reduction on the PCL-5 is considered the minimum reduction in severity of symptoms to constitute a clinically meaningful improvement [59–61]. A 4-point reduction on the EPDS indicates clinically meaningful reduction in depression symptoms [62].

### Ethics

The project was reviewed and approved by the medical center’s Institutional Review Board. Participants provided written, informed consent before completing the initial intake session. Study staff explained the study procedures, risks, benefits, and alternatives to patients, emphasizing that participation has no impact on their medical care. Participants were informed they could withdraw at any time and receive referrals for mental health treatment without penalty. Participants were also compensated for their time completing study measures as well as their travel and any parking expenses incurred with up to $140 in Target gift cards.

### Trustworthiness

All study therapists were either licensed clinical psychologists, clinical psychology PhD trainees, or Master’s level clinicians who completed a 12-h onsite NET training course with an expert trainer. The trainer continued to provide remote supervision for all cases reported in this paper. All researchers conducting this study had a background in the treatment of PTSD, including expertise in addressing sensitive clinical issues and mental health crisis management. The psychotherapy clinic where the study was conducted has a long-standing collaborative relationship with the department of obstetrics and gynecology. The study director (who also served as a study therapist) holds a joint faculty appointment in the department of obstetrics and gynecology.

Telehealth procedures using the Vidyo platform were informed by HIPAA, State and Federal guidelines. Vidyo maintains an information security governance policy that controls the confidentiality, integrity and availability of information handling, and includes a framework that prevents the misuse of information. As tele-services allow participants to log into a session from both secure private networks and unsecure public networks, the Vidyo platform protects again unauthorized access, third party hacking and voyeurism using encrypted token technology and transport layer security (TLS) certificate support. Vidyo has ensured appropriate technical protections are in place, in accordance with State and Federal guidelines, to detect, and as much as reasonably possible prevent, breach of confidentiality threats.

## Results

Over the six-month recruitment period, 208 pregnant patients completed the routine prenatal clinic screening for PTSD symptoms using the PCL-2, of which 41 screened positive (See Fig. 1 for participant flow diagram). Additionally, six patients were referred directly to the study by their prenatal care providers and four were referred once they had contacted the specialty psychotherapy clinic for treatment of perinatal distress. Together, referrals to the study totaled 51 pregnant patients. Of these, 24 completed the eligibility interview via telephone, 16 met criteria for inclusion, nine completed the informed consent process, and eight completed the full initial intake session and at least part of the NET treatment sessions. One participant terminated treatment prior to completing the intake session. Seven met eligibility criteria but did not attend the initial intake and informed consent session to enroll. One of these patients could not be reached to schedule the initial intake and informed consent process, and six scheduled the intake and informed consent session but did not attend. No patients who were eligible declined the program.

The results described in this case series include the eight pregnant participants who received at least four sessions of NET. The median number of lifetime traumatic events was 6.5 (range 1–16), the most common were interpersonal traumas, including childhood physical or sexual abuse, sexual or physical assault, or assault with a weapon (see Table 2). Traumatic childbirth was also common, with four participants reporting a previous traumatic childbirth experience, miscarriage, fetal death/stillbirth, and/or neonatal death.

**Table 2:** Counts of traumatic events endorsed by participants on life events checklist (LEC)

			Category Endorsed					
			Happened to Me	Witnessed it	Learned about it	Part of my job	Not sure	Doesn't apply
1. Natural disaster (e.g. flood)			1					7
2. Fire or explosion				1				7
3. Transportation accident (e.g. car accident)			4		2			3
4. Serious accident at work, home, or during recreational activity)								8
5. Exposure to toxic substance (e.g. radiation)			1			1		6
6. Physical assault (e.g. being attacked)			5		1			2
7. Assault with a weapon (e.g. being shot)			4		2			2
8. Sexual assault (e.g. rape)			3		1			4
9. Other unwanted or uncomfortable sexual experience			4		1			4
10. Combat or exposure to a war-zone (in the military or as a civilian)								8
11. Captivity (e.g. being kidnapped)			2					6
12. Life-threatening illness or injury			2	3	1		1	2
13. Severe human suffering			1		1	1		5
14. Sudden violent death (e.g. homicide)					3			5
15. Sudden accidental death			1		3		1	3
16. Serious injury, harm, or death you caused to someone else			1					7
17. Any other very stressful event or experience			6	1	1			2

*Note*: Participants could endorse more than one category per type of traumatic event so counts can be > 8

In the following sections, findings from this case series are structured according to the primary outcomes of interest: (1) feasibility of engagement and retention, (2) treatment expectations and acceptability of NET, and (3) treatment outcomes for symptoms of PTSD and depression from T1 to T3. Additionally, we report gestational weeks for each participant as they progressed through the treatment in order to examine the timing in pregnancy of when each woman engaged in and the length of time it took to complete treatment relative to the proposed 6-week treatment model.

### Feasibility

The overall proportion of pregnant patients who met eligibility criteria and initially agreed to participate that ultimately engaged in treatment was N = 9 (56%). Pregnant patients who were eligible but not yet consented were contacted multiple times by phone, email, and/or text, based on their preference. However, because the remaining seven participants could not be reached and did not follow up with study staff, we were unable to determine reasons for not engaging in treatment. In contrast, retention in treatment once engaged and completion of T3 assessment measures was high. Eight (89%) of the nine participants who began NET treatment completed at least 4 of the 6 sessions, considered the minimum dose for PTSD reduction for NET. Most participants experienced some alterations to the proposed 6-week treatment model in order for the treatment to flexibly meet participant needs and maintain engagement. All eight participants (100%) completed the T3 assessment measures.

#### Barriers to treatment

*Time to complete the intervention* Participants attended the initial intake session at any time between 18 and 30 weeks gestation (median gestational week = 22.5). A detailed timeline depicting delivery of treatment by gestational week is found in Table 3. The timeline reveals that, as a group, participants were generally well into their second trimesters when they began treatment. Also shown in Table 3, most participants experienced at least one gap of a week or more in between NET sessions, extending the overall duration of treatment beyond the proposed protocol of six weeks. The median number of weeks to complete four sessions was seven weeks (range 5–10 weeks). One participant who began treatment at 30 gestational weeks finished at 36 gestational weeks while another began treatment at 27 weeks and finished at 37 gestational weeks. This raised concerns for both therapists and the participants of how to maximize the likelihood of receiving at least the minimum number of sessions for clinical benefit prior to delivery when treatment engagement may become even more challenging and risk of postpartum distress may increase.

Regarding the structure of the NET treatment as outlined in Table 1, not all participants were able to complete the full initial intake session, consisting of the informed consent process, assessing the full lifetime trauma history and PTSD symptoms, psychoeducation about trauma and PTSD, and laying the lifeline, in one 2-h session. Table 3 also shows how these treatment components were divided across more than one session for two participants. Only one participant was able to complete the entire treatment (six weekly sessions including the initial intake session) within the proposed six-week treatment period. It is notable that this participant was not employed outside the home and had children enrolled in school fulltime.

*Schedule conflicts* Five of eight participants reported barriers due to schedule constraints, identifying factors such as employment, school, and prenatal appointments. One participant was two hours late to a session due to work delays and having to utilize public transportation during rush-hour traffic. Two participants reported lack of childcare was a barrier to attending planned sessions. In one case, the participant arrived at two sessions with two children (ages 1 and 5 years) and, with permission, a member of the study team supervised the children in another room during the exposure sessions. Flexibility on the part of study staff, therapists, and the study director enabled sessions to be rescheduled or extended and to hold sessions during evening hours as necessary, such as for one participant who lived 45 min from the clinic, worked full time during the day, and could not take time from work. In total, three participants’ sessions had to be rescheduled up to three times in order to be able to complete the treatment. One of these participants ultimately decided, in consultation with the study therapist, to shorten the treatment and begin telehealth sessions earlier than planned in order to enable her to complete an additional session rather than withdraw from treatment altogether.

*Variability of treatment across participants* The number of potential exposure sessions varied across participants due to the varying nature and extent of individual trauma histories. Moreover, despite all four therapists having received the same training and supervision from the same NET trainer, variations across therapy styles and approach occurred, which is not uncommon, even with manualized treatments. The median number of exposures was 5.5 (range = 3–6). Most participants completed a combination of stone and flower exposures. Given the brevity of treatment and the importance of processing traumas in order to reduce PTSD symptoms, priority was given to the traumatic events or stones, rather than flowers. The median number of stone exposures completed was 4.5 (range = 3–5). This meant that sometimes multiple exposures of different events were conducted in a single session with events processed in chronological order. Decisions regarding how many stone (or flower) to conduct and which events specifically was made between each therapist and participant, prioritizing focus on traumatic events underlying current PTSD symptoms (i.e., index trauma). Each exposure represented a unique event for all participants, meaning that exposures were not repeated for the same event. In cases of early childhood abuse/maltreatment, at least one childhood event was selected prior to processing more recent events. Three participants completed only stone exposures and no flowers. We noted that all three of these participants worked with the same study therapist.

Despite the high level of engagement of participants in completing the stone exposures, avoidance of trauma memories and discussing details of traumatic events is common. In our series of cases, avoidance manifested clinically in different ways, both explicitly and implicitly. One participant (ID 012) verbalized a strong desire not to discuss an event of childhood sexual abuse due to concern that her fetus would be negatively affected, whereas she did not express this fear about discussing previous neonatal death of twins born at 27 gestational weeks and another experience of intrauterine fetal death/stillbirth. The losses were of more proximal concern in the context of her current high-risk pregnancy and these were the events she identified as index traumas when reporting PTSD symptoms. In contrast, another participant (ID013) with 16 traumatic events described explicit interest in processing experiences of sexual abuse and assault. Nevertheless, she did evince avoidance while describing the details of the “hotspot” of sexual assault experiences, which manifested as rushing through the narration of the worst moments of the event (a common behavioral avoidance response), to which the therapist responded by asking her to repeat those parts of the event. Another example is how a participant (ID011), in contrast to the index trauma of a recent miscarriage, labeled earlier events as “small stones” as not worthy of examination, suggesting a reluctance to engage emotionally with earlier traumatic events. In this case the therapist encouraged conducting exposures of the “small” stones, and the two worst events were processed before the miscarriage.

Topics related to ongoing and current stressors emerged in the course of treatment that affected participants’ emotional engagement in the stone exposures. For example, two participants (ID002 and ID012) presented to sessions with significant ongoing stress related to employment, finances, and interpersonal disputes and appeared to struggle to focus on the content of the stone exposure. These concerns may or may not have been related to underlying avoidance. In all of these situations described, therapists emphasized the importance of overcoming avoidance as critical to overcoming PTSD, providing structure in eliciting the trauma narratives while validating present-focused concerns. Additionally, because the current study was conducted within a multi-disciplinary setting in which psychosocial supports and interventions were readily accessible, therapists facilitated referrals to these programs based on participant interest and expressed need.

*Conducting the telehealth sessions *Once participants had completed the initial four NET sessions in person in the psychotherapy clinic, the final two sessions were offered via secure telehealth platform using a smartphone app. Participants and therapists arranged an “appointment time” and they both logged on to the Vidyo platform’s virtual “therapy room” at the appointed time for their session. All participants were informed that a free device and data plan could be provided to them at no cost, however, none of the eight needed this service to participate. No difficulties were encountered with participants downloading the application and accessing the virtual therapy room. Some participants expressed concerns with connectivity such as losing the connection during session or poor quality of the connection, but sessions were ultimately completed. Only one participant and therapist during one session switched to a telephone to complete the exposure.

Multiple participants verbalized that accessing telehealth sessions from a location of their choice was a positive attribute of the treatment, enhancing their desire to participate in the study. Two participants (ID002 and ID011) indicated that while scheduling ease and reduced travel time made telehealth sessions favorable, beginning treatment in person helped them to develop a sense of trust in the therapist and confidence in NET. Participant concerns about privacy were carefully considered in each case, allowing for personalized and informed decisions about whether and how to use the telehealth. One participant, for example, expressed reticence about conducting an exposure session in the home shared with someone involved in the traumatic event. The therapist confirmed that there was no prior history of intimate partner violence in the relationship and that the participant’s safety was not at risk. Nevertheless, the participant chose to come to clinic for the exposure session, rather than use telehealth.

Another participant did not have access to childcare for her two year-old, and the exposure session was conducted with the child present, sitting on the participant’s lap and occasionally interacting with the clinician. As needed, the participant paused the session to care for the child but was able to engage with the exposure despite these mild distractions. Participants were generally engaged in the exposure sessions conducted via telehealth, and therapists did not report a significant difference from attending to the distractions that materialize in clinic settings (e.g., phones ringing, preoccupation with other tasks).

*Assessing safety *Participants were assessed for suicide risk, including ideations, means, intent, protective factors, and plans or thoughts of self-harm, at each session. The initial intake session included a full psychosocial history and assessment of current social relationships, allowing therapists to attend to risks of interpersonal violence that might arise at any point during treatment. As a group, participants represented a low to moderate risk group in terms of suicide risk. One participant (ID012) reported a history of multiple previous suicide attempts and expressed increasing feelings of hopelessness, helplessness, and severe coping impairment around the fourth session of NET. This seemed to be associated with extreme anxiety about the health of the current pregnancy (this participant had experienced multiple prior pregnancy losses). The therapist conducted a full safety and risk assessment, which led to temporary interruption of NET, consultation with the hospital’s psychiatric care team, and consideration of inpatient hospitalization. The participant was not acutely suicidal, and ultimately the distress improved with the support of the study therapist and the participants’ concurrent, long-term counselor. Once able to resume NET, this participant went on to complete two additional stone (trauma) exposures and one flower (positive event) exposure. In this case, the flower represented an experience of a live birth after she’d experienced multiple perinatal losses, which was particularly meaningful and empowering as she looked forward with a combination of hope and fear to the birth of another live, healthy infant. Another participant (ID002) reported an exacerbation of distress around the fourth NET session, related to an interpersonal conflict with her current partner. The participant’s obstetrician prescribed anti-depressant medication, and the participant reported improvement at the final session five weeks later.

### Acceptability

#### Pre-treatment expectations

As an assessment of confidence in treatment, we examined the number of participants who reported a 50% or higher chance of success in trauma recovery and treatment success. Most participants (7 out of 8) believed in their ability to heal from trauma and recover from PTSD (6 out of 8 participants). However, less than half felt confident that they would be successful in PTSD treatment (4 out of 8 participants endorsing the 50% threshold). Participants entering the treatment appeared to believe in their ability to heal from trauma and PTSD but had less confidence in PTSD treatment itself. The most common reason participants gave for beginning NET was to improve mental and physical health while pregnant. Participants noted minimal concerns about participating in NET (see Table 4 for all qualitative responses).

**Table 3:**
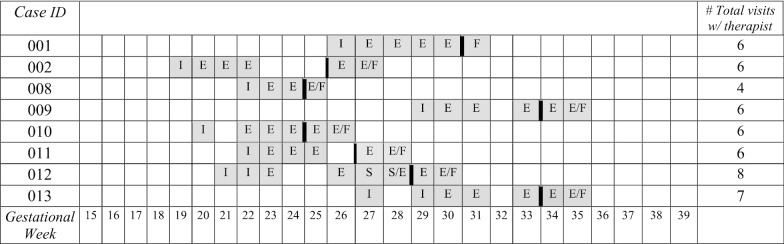
Delivery of NET Treatment to Participants by Gestational Week

*Note.* I = Initial intake session (informed consent, assess trauma history and symptoms, psychoeducation of trauma and PTSD, laying the lifeline); E = Exposure session; S = Stop procedure due to safety, complication, or adverse event; F = Final session; Where more than one label appears in a given week indicates multiple sessions in one week or cases in which an exposure was conducted in the final session; Solid vertical black line indicates start of telehealth sessions.

**Table 4:** Participants’ qualitative responses to expectations and experiences with NET

Case ID	Treatment Expectations		Treatment Experiences
	What are your reasons for participating in NET?	What are your concerns about participating in NET?	What did you like about the program? What would you change?
001	I feel it will help me deal with things I have never really addressed	That it will make me think even more about these past events	I like that we used both good and bad memory's and experiences Nothing I think it was great
002	I want to be happy again and feel better about myself and my family	That it won’t work	Everything Nothing
008	I know how I'm feeling isn't normal and I'm okay with that. I would like tools on how to deal with stressful situations better and how to feel okay. I don't want to feel sad or nervous	I'm not interested in medication	Being able to talk through my experiences and realize what I've been through was therapeutic. Being in a safe place to actualize and work through helped open up paths to healing Counseling for after
009	To let everything I been keeping inside of me out	Would it really help me better myself?	That I have finally spoke to someone about my pass that I would build inside me for years and having trouble in trusting anyone and letting them in. Thank you very much!! I would change nothing about it. It helped me relieve myself and stay positive about situation and change my whole mood of each day. Giving myself HAPPINESS
010	I want to be able to enjoy my pregnancy without fear and guilt	No concerns	The thing I like the most is looking back into my own memories to recognize those that changed me as a person I wish it would of last longer
011	I've never done counseling/therapy and was interested in the process. I thought it might be helpful	Finding time to come to treatment	I like the concept of making a narrative about my life history and going through all the major events. It shows you that there are a number of happy events in your life as well as some of the unhappy, challenging events. I like attaching a physical aspect to the life event like the flower or the rock. I like how the program was free Nothing right now
012	How to block my past	None	Story. The patience and genuine care the counselor provided Nothing I loved it
013	I am interested in this program to get the proper help I need to bring my baby into this world with no bad energy within me or around me	None	The counselor was very opened, nice, and patient Having more time

#### Treatment evaluation

On the post-treatment NET acceptability measure, seven out of the eight participants reported highest degree of acceptability. Every participant reported that they felt safe discussing traumatic events. Seven out of eight participants endorsed the belief that NET could provide relief for depressed mood, telling their story helped them to work through negative emotions, time needed for NET sessions is tolerable, and that they were helped by their NET counselor. The lowest scoring item on the acceptability measure was “*Six weeks of NET services is enough*”, indicating that some participants are interested in further psychotherapy.

Participants also completed a 5-item study-specific measure of perceived treatment benefit. On average, participants endorsed ‘Quite a bit’ to ‘Extremely’ for all questions concerning benefit, continuing use of skills, and behavior change. Participant responses to the free-response questions about what they liked/disliked are provided in Table 4.

### Treatment outcomes

Participants showed clinically meaningful improvements in PTSD and depressive symptom scores from T1 to T3. Between the eligibility interview and TI, three of the eight participants exhibited reduced PTSD symptom severity to subthreshold scores (< 33 on the PCL-5) and continued to show improved symptoms over the course of treatment. The remaining five participants demonstrated at least a 10-point reduction in severity of symptoms from T1 to T3. In total, seven of eight participants (88%) reported a minimum 10-point reduction on the PCL-5 from T1 to T3. All eight participants (100%) had a minimum 4-point reduction on the EPDS from T1 to T3.

## Discussion

The results described in this report demonstrate promising findings for the first exploration into exposure therapy treatment of PTSD during pregnancy. Results suggest the majority of NET participants completed an adequate number of treatment sessions despite significant barriers, expected they would be able to recover from past traumas, were satisfied with the treatment, and found NET to be acceptable and relevant to their emotional needs for post-trauma recovery. Qualitatively, the participants described treatment as helpful, some stating they wished for more. Further, participants exhibited clinically significant reductions in posttraumatic stress and depressive symptoms.

### Evaluation of NET feasibility, acceptability, and treatment outcomes

Our findings suggest it is feasible to provide brief evidence-based PTSD treatment during pregnancy, albeit with some challenges to treatment engagement. Findings are encouraging given the clinical need: Approximately 25% of pregnant patients presenting to the obstetrics and gynecology clinics at our academic medical center screened positive for PTSD and almost none receive treatment for their distress. The positive screen rate reflects the significant trauma load of pregnant individuals in our clinics as well as their significant social disadvantage, underscoring the importance of addressing the large gaps in mental health treatment for PTSD in the perinatal period.

Treatment retention for the eight participants in this case series was also highly encouraging, although the majority of pregnant participants identified with trauma-related distress were either not reachable to complete the eligibility interview or chose not to engage in treatment. The experiences of the individuals described in this case series is therefore not representative of the perinatal population as a whole. More research is needed to investigate reasons that govern whether an individual chooses to pursue treatment or not as well as to investigate strategies to reduce stigma and overcome avoidance of addressing past traumas. Nevertheless, for the nine participants who initiated treatment, all but one was able to complete at least 4 sessions of NET in under 10 weeks. Further, seven of the eight participants completed the full 6 sessions of NET. The one participant who dropped out reported that she did not have the time to complete study activities due to other life demands and priorities. This preliminary data is highly encouraging for exploration of offering NET as a frontline treatment for trauma-exposed pregnant individuals with PTSD.

Results of this case series also highlights the need to be mindful of strategies to enhance engagement and retention in treatment to reduce barriers once treatment is initiated. Whether these barriers can be attributed to trauma avoidance, pregnancy-specific challenges (i.e., physiological symptoms of pregnancy, prenatal appointment burden), or lack of resources (e.g., transportation, flexible work schedule, childcare) was not specifically addressed in this study. However, the experience of offering and conducting NET treatment with this small group shed light on questions that could be systematically investigated. For example, it is important to consider strategies that focus on balancing demands of multiple roles and responsibilities (e.g., caring for older children, work) with minimal social support and significant financial stress. Persistence on the part of the participant combined with flexibility of treatment providers was necessary for all participants to complete treatment, although almost every participant cancelled or rescheduled a session at least once. The study found that week-to-week schedule changes were the norm, not the exception, especially for individuals with high stress (e.g., inflexible employer) and multiple demands on their time (e.g., caring for other children). Thus, additional supports may be necessary to enable individuals to prioritize treatment without sacrificing other responsibilities.

In an effort to mitigate some of the challenges described, the current study explored use of telehealth for location convenience and travel reduction. This approach worked generally well in this small group. We had anticipated lack of access to a smartphone device or data plan to allow telehealth sessions but this was not the case for these eight participants. More pressing was the concern for lack of privacy and space to ensure participants would be able to delve into the details of deeply personal and sensitive experiences without third party interruption or observation. As challenging as it may be to keep regular, scheduled appointments, physically go to a clinic, and make arrangements for childcare, some participants expressed that the coming to clinic afforded them a sense of safety, security, and privacy not available elsewhere. For others, being at home did not solve the need to find childcare if there were other young children in the home requiring supervision.

Notwithstanding the challenges, this group of eight participants was highly satisfied with NET. Additionally, self-reported PTSD and depression symptoms improved as treatment progressed following a similar pattern to other evidence-based PTSD treatments such as Cognitive Processing Therapy (CPT) [63] and Prolonged Exposure (PE) [14]. An important advantage of NET, however, is the association with symptom-reduction in a shorter timeframe and with lower dropout rates, on average, compared to other exposure-based treatments [32]. NET may be a preferable option for groups that struggle with completion of therapeutic work outside of the clinical sessions or those interested in shorter-term treatment [32]. Efficacy, effectiveness, and implementation of multiple PTSD treatments will need to be examined in perinatal samples in order to explore some of these questions.

## Limitations

This study had several limitations. Only a very small group received treatment, limiting what can be extrapolated on a larger scale. Almost half of eligible participants signaled interest but did not attend the initial intake session, highlighting a need to know what factors influence whether a pregnant individual with active trauma symptoms ultimately engages in treatment, and indeed, whether similar factors influence degree of avoidance during treatment in those who do engage. However, because inclusion criteria were wide, the sample was diverse in terms of age, parity, racial/ethnic background, trauma type and severity. The design did not include a control group preventing comparisons to pregnant individuals with PTSD who did not receive treatment. The current study also examined a limited model of treatment – only six sessions – in order to maximize likelihood that active treatment could be completed within a short timeframe before childbirth. Therefore, we do not know the outcomes of a treatment model where the number of sessions is flexible or additional supports are added following the exposure therapy to maintain treatment gains (although referrals to additional psychotherapy/counseling were provided).

Given that NET was the only treatment offered to participants and the data are based on only a small case series, results should be interpreted with caution as to the generalizability of findings to other pregnant populations and other treatment settings. Future research will be important to examine a range of treatment models and approaches and to understand factors that shape whether a particular model is a good fit for a particular group. Next, the treatment team included four different therapists so there could be differences in treatment outcomes across therapists not captured in the current study. However, all therapists have prior training and experience with perinatal individuals with trauma histories and all therapists participated in the same onsite NET training and received individual and group supervision from the same trainer to ensure adherence to the NET model. Finally, we have not conducted long-term follow-up after 1-month postpartum so it is unknown whether symptoms remained improved, worsened, or stayed the same after treatment. All participants were provided with coordinated referrals to our mental health services throughout the course of the study to ensure access to ongoing treatment where needed or desired.

## Conclusion

This feasibility study and case series analysis provided preliminary support for the usefulness of NET for pregnant individuals with PTSD and provided valuable insights into aspects of the treatment approach that require further investigation, development, and evaluation, particularly compared with other treatment modalities. In the case series, participants demonstrated improvements in PTSD symptoms from pre- to post-treatment and did not report any adverse events. Results support the viability of exposure therapy using NET to treat PTSD during pregnancy, with consideration for the fact that results of this case series are based on only a small group of participants. Future research should continue to explore the use of NET in perinatal populations, as well as to examine how reduction of PTSD symptoms prenatally may ultimately improve perinatal health outcomes.

## Data Availability

The datasets analyzed during the current study are available from the corresponding author on reasonable request and subject to Institutional Review Board approval.

## References

[1] Yildiz PD, Ayers S, Phillips L (2017). The prevalence of posttraumatic stress disorder in pregnancy and after birth: a systematic review and meta-analysis. J Affect Disord.

[2] Seng JS, Low LK, Sperlich M, Ronis DL, Liberzon I (2011). Post-traumatic stress disorder, child abuse history, birthweight and gestational age: a prospective cohort study. BJOG.

[3] Yonkers KA, Blackwell KA, Glover J, Forray A (2014). Antidepressant use in pregnant and postpartum women. Annu Rev Clin Psychol.

[4] Shaw JG, Asch SM, Kimerling R, Frayne SM, Shaw KA, Phibbs CS (2014). Posttraumatic stress disorder and risk of spontaneous preterm birth. Obstet Gynecol.

[5] Shaw JG, Asch SM, Katon JG, Shaw KA, Kimerling R, Frayne SM (2017). Post-traumatic stress disorder and antepartum complications: A novel risk factor for gestational diabetes and preeclampsia. Paediatr Perinat Epidemiol.

[6] Nillni YI, Mehralizade A, Mayer L, Milanovic S (2018). Treatment of depression, anxiety, and trauma-related disorders during the perinatal period: A systematic review. Clin Psychol Rev.

[7] Sperlich M, Seng JS, Rowe H, Cameron H, Harris A, McCracken A, Rauch SA, Bell SA (2011). The Survivor Moms’ Companion: feasibility, safety, and acceptability of a posttraumatic stress specific psychoeducation program for pregnant survivors of childhood maltreatment and sexual trauma. Int J Childbirth.

[8] Upshur CC, Wenz-Gross M, Weinreb L, Moffitt JJ (2016). Using prenatal advocates to implement a psychosocial education intervention for posttraumatic stress disorder during pregnancy: feasibility, care engagement, and predelivery behavioral outcomes. Women's Health Issues.

[9] Zlotnick C, Capezza NM, Parker D (2011). An interpersonally based intervention for low-income pregnant women with intimate partner violence: a pilot study. Arch Womens Ment Health.

[10] Rowe H, Sperlich M, Cameron H, Seng J (2014). A quasi-experimental outcomes analysis of a psychoeducation intervention for pregnant women with abuse-related posttraumatic stress. J Obstet Gynecol Neonatal Nurs.

[11] Weinreb L, Wenz-Gross M, Upshur C (2018). Postpartum outcomes of a pilot prenatal care-based psychosocial intervention for PTSD during pregnancy. Arch Womens Ment Health.

[12] Seng JS, Sperlich M, Rowe H, Cameron H, Harris A, Rauch SA, Bell SA (2011). The survivor Moms’ companion: open pilot of a posttraumatic stress specific psychoeducation program for pregnant survivors of childhood maltreatment and sexual trauma. Int J Childbirth.

[13] Schauer M, Schauer M, Neuner F, Elbert T (2011). Narrative exposure therapy: a short-term treatment for traumatic stress disorders.

[14] Foa E, Hembree E, Rothbaum BO. Prolonged exposure therapy for PTSD: Emotional processing of traumatic experiences therapist guide. Oxford University Press, Oxford; 2007

[15] Rauch SA, Eftekhari A, Ruzek JI (2012). Review of exposure therapy: A gold standard for PTSD treatment. J Rehabil Res Dev.

[16] Rosen D, Seng JS, Tolman RM, Mallinger G (2007). Intimate partner violence, depression, and posttraumatic stress disorder as additional predictors of low birth weight infants among low-income mothers. J Interpers Violence [Internet].

[17] Lev-Wiesel R, Chen R, Daphna-Tekoah S, Hod M (2009). Past traumatic events: Are they a risk factor for high-risk pregnancy, delivery complications, and postpartum posttraumatic symptoms?. J Womens Health [Internet].

[18] Seng JS, Kohn-Wood LP, McPherson MD (2011). Disparity in posttraumatic stress disorder diagnosis among African American pregnant women. Arch Womens Ment Health.

[19] Dailey DE, Humphreys JC, Rankin SH, Lee KA (2011). An exploration of lifetime trauma exposure in pregnant low-income African American women. Matern Child Health J.

[20] Belle D, Doucet J (2003). Poverty, inequality, and discrimination as sources of depression among US women. Psychol Women Q.

[21] Ennis NE, Hobfoll SE, Schröder KE (2000). Money doesn't talk, it swears: How economic stress and resistance resources impact inner-city women's depressive mood. Am J Community Psychol.

[22] Schumm JA, Stines LR, Hobfoll SE, Jackson AP (2005). The double-barreled burden of child abuse and current stressful circumstances on adult women: the kindling effect of early traumatic experience. J Trauma Stress.

[23] Davis RG, Ressler KJ, Schwartz AC, Stephens KJ, Bradley RG (2008). Treatment barriers for low-income, urban African Americans with undiagnosed posttraumatic stress disorder. J Trauma Stress [Internet].

[24] Robjant K, Fazel M (2010). The emerging evidence for narrative exposure therapy: a review. Clin Psychol Rev.

[25] Schaal S, Elbert T, Neuner F (2009). Narrative exposure therapy versus interpersonal psychotherapy. Psychother Psychosom.

[26] Neuner F, Onyut PL, Ertl V, Odenwald M, Schauer E, Elbert T (2008). Treatment of posttraumatic stress disorder by trained lay counselors in an African refugee settlement: a randomized controlled trial. J Consult Clin Psychol.

[27] Ertl V, Pfeiffer A, Schauer E, Elbert T, Neuner F (2011). Community-implemented trauma therapy for former child soldiers in Northern Uganda: A randomized controlled trial. JAMA.

[28] Cienfuegos AJ, Monelli C (1983). The testimony of political repression as a therapeutic instrument. Am J Orthopsychiatry.

[29] McQueen A, Kreuter MW, Kalesan B, Alcaraz KI (2011). Understanding narrative effects: the impact of breast cancer survivor stories on message processing, attitudes, and beliefs among African American women. Health Psychol.

[30] Goddu AP, Raffel KE, Peek ME (2015). A story of change: the influence of narrative on African-Americans with diabetes. Patient Educ Couns.

[31] Akinyela MM (2005). Testimony of hope: African centered praxis for therapeutic ends. J Syst Ther.

[32] Mørkved N, Hartmann K, Aarsheim LM, Holen D, Milde AM, Bomyea J (2014). A comparison of narrative exposure therapy and prolonged exposure therapy for PTSD. Clin Psychol Rev.

[33] Volpe EM, Quinn CR, Resch K, Sommers MS, Wieling E, Cerulli C (2017). Narrative exposure therapy: a proposed model to address intimate partner violence-related PTSD in parenting and pregnant adolescents. Fam Community Health.

[34] Volpe EM, Quinn CR, Resch K, Douglas V, Cerulli C (2017). Assessing the feasibility and acceptability of narrative exposure therapy to address IPV-related mental health in parenting and pregnant adolescents. J Fam Violence.

[35] Backhaus A, Agha Z, Maglione ML, Repp A, Ross B, Zuest D, Rice-Thorp NM, Lohr J, Thorp SR (2012). Videoconferencing psychotherapy: A systematic review. Psychol Serv.

[36] Gros DF, Morland LA, Greene CJ, Acierno R, Strachan M, Egede LE, Tuerk PW, Myrick H, Frueh BC (2013). Delivery of evidence-based psychotherapy via video telehealth. J Psychopathol Behav Assess.

[37] Morland LA, Hynes AK, Mackintosh MA, Resick PA, Chard KM (2011). Group cognitive processing therapy delivered to veterans via telehealth: A pilot cohort. J Trauma Stress.

[38] Yuen EK, Gros DF, Price M, Zeigler S, Tuerk PW, Foa EB, Acierno R (2015). Randomized controlled trial of home-based telehealth versus in-person prolonged exposure for combat-related PTSD in veterans: preliminary results. J Clin Psychol.

[39] Franklin CL, Cuccurullo LA, Walton JL, Arseneau JR, Petersen NJ (2017). Face to face but not in the same place: a pilot study of prolonged exposure therapy. J Trauma Dissoc.

[40] Taft CT, Resick PA, Watkins LE, Panuzio J (2009). An investigation of posttraumatic stress disorder and depressive symptomatology among female victims of interpersonal trauma. J Fam Violence.

[41] Blevins CA, Weathers FW, Davis MT, Witte TK, Domino JL (2015). The Posttraumatic Stress Disorder Checklist for DSM-5 (PCL-5): Development and initial psychometric evaluation. J Trauma Stress.

[42] Lang AJ, Stein MB (2005). An abbreviated PTSD checklist for use as a screening instrument in primary care. Behav Res Ther.

[43] Neuner F, Schauer M, Klaschik C, Karunakara U, Elbert T (2004). A comparison of narrative exposure therapy, supportive counseling, and psychoeducation for treating posttraumatic stress disorder in an African refugee settlement. J Consult Clin Psychol.

[44] Zang Y, Hunt N, Cox T (2013). A randomised controlled pilot study: the effectiveness of narrative exposure therapy with adult survivors of the Sichuan earthquake. BMC Psychiatry.

[45] Vidyo Connect. (2005). Vidyo Inc. (Version 20.1.1.2968) [Mobile application software]. http://vidyo.com

[46] Harris PA, Taylor R, Thielke R, Payne J, Gonzalez N, Conde JG (2009). Research electronic data capture (REDCap)—a metadata-driven methodology and workflow process for providing translational research informatics support. J Biomed Inform.

[47] Harris PA, Taylor R, Minor BL, Elliott V, Fernandez M, O'Neal L, McLeod L, Delacqua G, Delacqua F, Kirby J, Duda SN (2019). The REDCap consortium: Building an international community of software platform partners. J Biomed Inform.

[48] Posner K, Brown GK, Stanley B, Brent DA, Yershova KV, Oquendo MA (2011). The Columbia-Suicide Severity Rating Scale: Initial validity and internal consistency findings from three multisite studies with adolescents and adults. Am J Psychiatry.

[49] Na PJ, Yaramala SR, Kim JA, Kim H, Goes FS, Zandi PP, Voort JL, Sutor B, Croarkin P, Bobo WV (2018). The PHQ-9 Item 9 based screening for suicide risk: a validation study of the Patient Health Questionnaire (PHQ)− 9 Item 9 with the Columbia Suicide Severity Rating Scale (C-SSRS). J Affect Disord.

[50] Szpunar MJ, Crawford JN, Baca SA, Lang AJ (2020). Suicidal ideation in pregnant and postpartum women veterans: an initial clinical needs assessment. Mil Med.

[51] Weathers FW, Bovin MJ, Lee DJ, Sloan DM, Schnurr PP, Kaloupek DG (2018). The Clinician-Administered PTSD Scale for DSM–5 (CAPS-5): Development and initial psychometric evaluation in military veterans. Psychol Assess.

[52] Marshall RD, Lewis-Fernandez R, Blanco C, Simpson HB, Lin SH, Vermes D, Garcia W, Schneier F, Neria Y, Sanchez-Lacay A, Liebowitz MR (2007). A controlled trial of paroxetine for chronic PTSD, dissociation, and interpersonal problems in mostly minority adults. Depress Anxiety.

[53] Forray A, Mayes LC, Magriples U, Epperson CN (2009) Prevalence of post-traumatic stress disorder in pregnant women with prior pregnancy complications. J Matern Fetal Neo 22(6):522–527. 10.1080/14767050902801686 PMC4109276 19488936

[54] Gelaye B, Zheng Y, Medina-Mora ME, Rondon MB, Sánchez SE, Williams MA (2017). Validity of the posttraumatic stress disorders (PTSD) checklist in pregnant women. BMC Psychiatry.

[55] Baas MA, Stramrood CA, Dijksman LM, de Jongh A, Van Pampus MG (2017). The OptiMUM-study: EMDR therapy in pregnant women with posttraumatic stress disorder after previous childbirth and pregnant women with fear of childbirth: design of a multicentre randomized controlled trial. Eur J Psychotraumatol.

[56] Cox JL, Holden JM, Sagovsky R (1987). Detection of postnatal depression: development of the 10-item Edinburgh Postnatal Depression Scale. Brit J Psychiat.

[57] Wisner KL, Parry BL, Piontek CM (2002). Postpartum depression. N Engl J Med.

[58] Quinn, CR. Net acceptability and feasibility questionnaire. 2015. Personal communication, obtained from creator.

[59] Jacobson NS, Truax P (1991). Clinical significance: a statistical approach to defining meaningful change in psychotherapy research. J Consult Clin Psychol.

[60] Clapp JD, Kemp JJ, Cox KS, Tuerk PW (2016). Patterns of change in response to prolonged exposure: implications for treatment outcome. Depress Anxiety.

[61] National Center for PTSD. Using the PTSD Checklist for DSM-5 (PCL-5). 2015. https://www.ptsd.va.gov/professional/assessment/adult-sr/ptsd-checklist.asp.

[62] Matthey S (2004). Calculating clinically significant change in postnatal depression studies using the Edinburgh Postnatal Depression Scale. J Affect Disord.

[63] Resick PA, Schnicke MK (1992). Cognitive processing therapy for sexual assault victims. J Consult Clin Psychol.

